# Quantitative Evaluation of Toxic Polyglycine Biosynthesis and Aggregation in Cell Models Expressing Expanded CGG Repeats

**DOI:** 10.3389/fgene.2018.00216

**Published:** 2018-06-19

**Authors:** Magdalena Derbis, Patryk Konieczny, Agnieszka Walczak, Michal Sekrecki, Krzysztof Sobczak

**Affiliations:** Department of Gene Expression, Institute of Molecular Biology and Biotechnology, Adam Mickiewicz University, Poznań, Poland

**Keywords:** FXTAS, FXS premutation, CGG repeat expansion, polyglycine, RAN translation

## Abstract

Fragile X-associated tremor/ataxia syndrome (FXTAS) is a late-onset neurodegenerative disorder caused by expanded CGG (CGG^exp^) trinucleotides in the 5′UTR of the *FMR1* gene encoding fragile X mental retardation protein (FMRP). The patients, with the number of the repeats ranging from 55 to 200, show specific manifestation of clinical symptoms that include intention tremor, gait ataxia, cognitive deficits, and brain atrophy. Accumulation of toxic polyglycine (FMRpolyG), a by-product of the CGG^exp^ repeat-associated non-ATG (RAN) translation, is considered to be one of the main factors triggering neurodegenerative processes in FXTAS patients. Nevertheless, the nature of the FMRpolyG-induced cell damage, especially in the context of its soluble and inclusion-associated forms, is still elusive. Targeting either biosynthesis, cellular stability or aggregation capacity of toxic FMRpolyG could be considered as a potential therapeutic strategy for FXTAS. Therefore, we tested a variety of quantitative methods based on forced expression of genetic constructs carrying CGG^exp^ repeats in the context of the *FMR1* 5′UTR fused to *GFP, mCherry* or Firefly luciferase gene in or out of frame to the polyglycine encoding sequence. We show that FMRpolyG translation either from native or an AUG-induced start codon as well as the translation yield of the FMRP open reading frame equivalent located downstream of the CGG^exp^ element can be effectively estimated using fluorescence microscopy, flow cytometry or luciferase assay. We also quantitatively estimated soluble fraction and insoluble form of FMRpolyG aggregated in foci using an electrophoretic separation of cell lysates and fluorescence microscopy, respectively. Importantly, we show that dependent on a fusion tag, FMRpolyG has a different potential for aggregate formation. Our established protocols enable sensitive tracking of FMRP and FMRpolyG quantitative and qualitative changes after treatment with potential therapeutic agents for FXTAS. Furthermore, they can be modified for application to other RAN translation- and aggregation-related diseases.

## Introduction

Fragile X-associated tremor/ataxia syndrome (FXTAS) is a late-onset neurodegenerative disorder caused by expanded CGG (CGG^exp^) trinucleotides in the 5′-untranslated region (5′UTR) of the *FMR1* gene (Hagerman and Hagerman, 2016; Boivin et al., 2017). The affected individuals start to suffer from intention tremor, cerebellar ataxia, neuropathic pain, parkinsonian features and cognitive deficits usually in their fifties or sixties (Jacquemont et al., 2003; Leehey et al., 2007). Magnetic resonance imaging reveals characteristic alterations in the brain that include sites of focal damage, white matter disease and the overall organ atrophy. Intranuclear ubiquitin-positive inclusions in both astrocytes and neurons are another distinctive feature of the disease (Greco et al., 2002; Greco et al., 2006). As the *FMR1* gene is located on the X chromosome, the male carriers are mostly affected, however the predominance is incomplete, with the current estimate of 16–20% females and 40–75% males to develop FXTAS (Seltzer et al., 2012; Tassone et al., 2012; Hunter et al., 2014). The mutation itself occurs with lower frequency in men (1:430–850) than in women (1:150–300). Despite the late onset of the full blown FXTAS symptoms, the carriers show manifestation of a milder phenotype earlier (Hagerman and Hagerman, 2016), including fragile X-associated primary ovarian insufficiency in females (FXPOI, Sullivan et al., 2005).

FXTAS belongs to a heterogeneous group of diseases caused by expansion of a microsatellite motif (Ciesiolka et al., 2017; Hannan, 2018). Depending on the site of the mutation in a gene, number of the repeats, and the repeated sequence, the toxicity can be exerted either by abrogation of protein expression, sequestration of specific proteins on the mutant RNA, or translation of additional, harmful peptides and proteins. The current state of knowledge indicates that the neuronal death observed in FXTAS patients is directly linked to the non-AUG (RAN) translated polyglycine (FMRpolyG), generated from the *FMR1* transcript adjacently to fragile X mental retardation protein (FMRP) (Todd et al., 2013; Buijsen et al., 2014; Hukema et al., 2015; Buijsen et al., 2016; Sellier et al., 2017). Particularly, Sellier et al. (2017) showed that upon mutant gene expression, FMRpolyG forms foci, binds LAP2beta, and disrupts the nuclear envelope, which precedes the neuronal death in patient cells. As the initiating weak ACG and GUG start codons are located before the CGG repeats (Kearse et al., 2016; Sellier et al., 2017), shorter FMRpolyG peptides are probably also produced in unaffected individuals, however, neurons seem to efficiently remove them, as no toxic inclusions are observed. Mutated *FMR1* mRNA can also exert adverse effects by itself, as several proteins such as a splicing regulator SAM68, or microRNA processing DROSHA and DGCR8, have been found sequestered on CGG^exp^ tracts (Sellier et al., 2010; Sellier et al., 2013; Glineburg et al., 2018). Nevertheless, it is important to stress that the expanded repeats alone did not cause cell death upon abrogation of FMRpolyG expression (Sellier et al., 2017).

FXTAS develops in individuals that contain from 55 to 200 CGG repeats in the 5′UTR of the *FMR1* gene. Intriguingly, upon longer expansion (more than 200 repeats), the transcription is shut down due to promoter hypermethylation and in affected individuals a disease called Fragile X syndrome (FXS) manifests, usually before the second year of age, although first symptoms can be apparent in early infancy (Hagerman et al., 2017). The specific symptoms of FXS include alterations in physical appearance, intellectual disability as well as autism that presumably result from the underlying changes in connective tissue and abnormalities in synaptic plasticity (Darnell and Klann, 2013; Nelson et al., 2013). Whole-transcriptome studies showed that FMRP takes part in regulation of site-specific translation in neurons (Darnell et al., 2011). Bearing in mind that FMRpolyG and FMRP are generated from the same transcript, albeit in different reading frames, and that production of FMRpolyG as well as abrogation of FMRP expression results in pathology has important implications and should be taken in mind while designing a successful therapeutic approach against FXTAS (Yang et al., 2015).

A therapeutically relevant decrease of the aggregated in foci FMRpolyG could be achieved by blocking FMRpolyG translation in the mutant *FMR1* transcript. One should consider, however, parallel downregulation of FMRP that could potentially turn pathogenic. As an alternative, lowering the stability or aggregation of FMRpolyG could also be considered as a valuable therapeutic option. In the present study, we aimed at selecting methods that could be potentially used to study therapeutic potential of compounds that would either target mutated *FMR1* RNA or the aggregation/stability of FMRpolyG.

## Materials and Methods

### Constructs

ATG(CGG^exp^)-*GFP*(+1) and 5′(CGG^exp^)-*GFP*(+1) (Addgene #63091) constructs were a kind gift from N. Charlet-Berguerand (see also Sellier et al., 2017). Briefly, 5′(CGG^exp^)-*GFP*(+1) contains the 5′UTR of the *FMR1* gene that contains 99 CGG repeats and is fused to *eGFP* sequence. Both proteins, polyglycine (FMRpolyG) and GFP are expressed as a fusion protein (FMRpolyG-GFP). In ATG(CGG^exp^)-*GFP*(+1), the weak ACG start codon is replaced for a strong ATG. In addition, the 5′ part of the *FMR1* 5′UTR, upstream the start codon, is missing from the construct. 5′(CGG^exp^)-*mCherry*(0) construct was derived from 5′(CGG^exp^)-*GFP*(+1). *mCherry* sequence was amplified from pmCherry_a_tubulin_IRES_puro2 (Addgene #21043) with (*F1*/*R1*) primers with CloneAmp HiFi PCR Premix kit (Takara Bio United States) according to manufacturer’s instructions. After digestion with AvrII and XbaI, purified and digested *mCherry* PCR product was ligated instead of *eGFP* sequence with digested and dephosphorylated construct backbone (CIAP, Thermo Fisher Scientific). The sequence was confirmed by Sanger sequencing. ATG-*luc2* was obtained from pmirGLO (Promega) by digestion with NheI and XbaI, ligation and insertion of annealed oligos into a HindIII site in front of *luc2* as previously published (Konieczny et al., 2017). ATG(CGG^exp^)-*luc2*(+1) was generated in a few sequential steps. First, a fragment containing 99 CGG repeats from NheI and XhoI ATG(CGG^exp^)-*GFP*(+1) was cloned into NheI and XhoI digested ATG-*luc2*. The obtained construct was then digested with EcoRI and XhoI and ligated to annealed oligos (*F2*/*R2*) in order to generate the final plasmid from which FMRpolyG and Firefly luciferase would be expressed as a fusion protein (FMRpolyG-Firefly). 5′(CGG^exp^)-*luc2*(0) is a product of ligation of a NheI digested and dephosphorylated ATG-*luc2* construct and an insert containing CGG repeats from NheI digested 5′(CGG^exp^)-*GFP*(+1) construct. FMRpolyG and Firefly luciferase are expressed as two independent proteins from this construct. To obtain ATG(CGG^exp^)-*mCherry*(+1) construct, *mCherry* sequence was amplified using *F3*/*R3* primers with KAPA HiFi DNA polymerase (Kapa Biosystems) on pmCherry_a_tubulin_IRES_puro2 construct template. The purified PCR product was digested with XhoI and XbaI and cloned in between XhoI and XbaI sites of ATG(CGG^exp^)-*GFP*(+1) construct. All the ligations were performed with T4 DNA ligase (Thermo Fisher Scientific). 5′(CGG^exp^)-*GFP*(+1) constructs with ∼20 and ∼50 CGG repeats (Supplementary Figure S3B) were obtained as a result of the repeat instability during the bacterial culture growth. The CGG repeat number was estimated following NheI digestion.

### Cell Culture

COS7 cell line was grown in a high glucose DMEM medium with L-Glutamine (Lonza) supplemented with 10% fetal bovine serum (Sigma) and 1% antibiotic/antimycotic (Sigma). Control fibroblast line (C0603, CGG^norm^/-) and two fibroblast lines obtained from FXTAS patients, FX11-02 with one mutant and one normal allele (CGG^norm^/CGG^exp^) and WC26 with two mutant alleles (CGG^exp^/CGG^exp^) were a kind gift from A. Bhattacharyya (see also Rovozzo et al., 2016). Fibroblast cell lines were grown in EMEM medium (Lonza) supplemented with 15% fetal bovine serum (Sigma), 1% MEM non-essential amino acids (Thermo Fisher Scientific) and 1% antibiotic/antimycotic (Sigma). All cell lines were grown at 37°C in a humidified incubator containing 5% CO_2_.

### Plasmid Delivery

**FIGURE 1:**
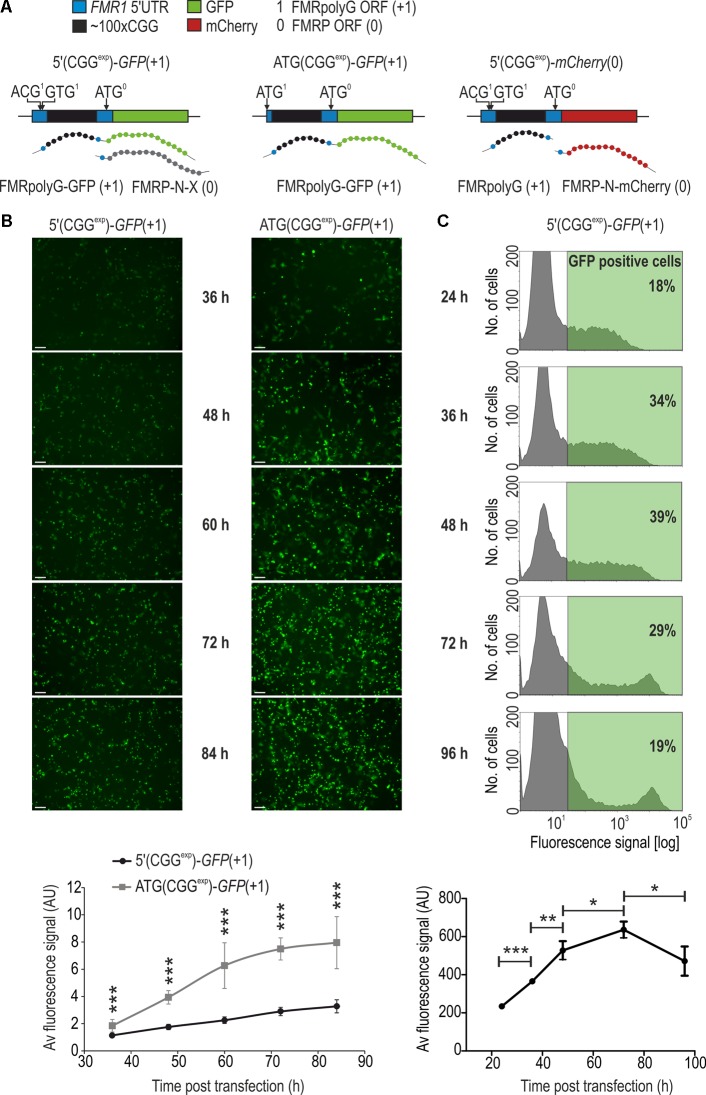
Fluorescence microscopy and flow cytometry are sensitive and informative tools for quantitative evaluation of RAN translation product, FMRpolyG-GFP. **(A)** Schematic representation of genetic constructs used in fluorescence microscopy, flow cytometry and FL-SDS-PAGE. Delivery of 5′(CGG^exp^)-*GFP*(+1) or ATG(CGG^exp^)-*GFP*(+1) leads to biosynthesis of FMRpolyG-GFP as a result of RAN translation from near-cognate ACG or GUG start codons or canonical translation from ACG to AUG mutated start codon, respectively. An additional protein, FMRP-N-X, can be generated from the FMRP native start codon of 5′(CGG^exp^)-*GFP*(+1). Administration of 5′(CGG^exp^)-*mCherry*(0) leads to biosynthesis of two separate proteins, non-tagged FMRpolyG and FMRP-N-mCherry generated from the FMRP-specific AUG start codon. **(B)** Fluorescence microscopy of COS7 cells 36, 48, 60, 72, or 84 h following transfection with 5′(CGG^exp^)-*GFP*(+1) or ATG(CGG^exp^)-*GFP*(+1). Images were taken from the same wells at different time points post transfection. Quantification of the average fluorescence signal is shown in the lower panel (*n* = 10). Scale bar, 500 μm. **(C)** Histograms showing distribution of the FMRpolyG-GFP signal in COS7 cells 24, 36, 48, 72, and 96 h after 5′(CGG^exp^)-*GFP*(+1) delivery and flow cytometry. In the lower panel, quantification of the average fluorescence signal after exclusion of dead cells is shown (*n* = 3). The threshold for GFP positive cells was set based on the signal obtained for non-transfected cells. For each sample ∼5000 GFP-positive cells were analyzed. Note that the percentage of GFP-positive cells reached maximum 48 h post transfection (39%) while the mean fluorescence signal was highest 72 h post transfection. In the following time points, a decrease in these values was observed as a consequence of cell division and cell death.

**FIGURE 2:**
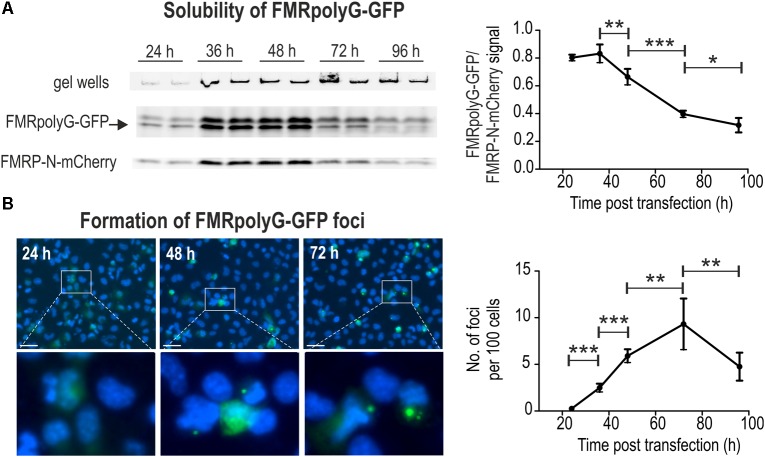
FL-SDS-PAGE and fluorescence microscopy allow for precise quantification of soluble and aggregated in foci forms of FMRpolyG-GFP. **(A)** FL-SDS-PAGE analyses of soluble fractions of FMRpolyG-GFP and FMRP-N-mCherry in cell lysates prepared 24, 36, 48, 72, and 96 h after co-transfection with 5′(CGG^exp^)-*GFP*(+1) and 5′(CGG^exp^)-*mCherry*(0). Fluorescence detected in gel wells is characteristic for FMRpolyG-GFP positive samples (invisible in case of other GFP-fusion proteins or GFP alone; not shown) and comes from a fraction of undissolved protein aggregates. The FMRpolyG-GFP fluorescence signal shown on the graph was normalized to FMRP-N-mCherry signal (*n* = 4). Note that after 36 h solubility of FMRpolyG-GFP decreases significantly in favor of the insoluble form of this protein. The FMRpolyG-GFP band was additionally identified based on western blotting with anti-FMRpolyG antibodies (see Supplementary Figure S3). **(B)** Fluorescence microscopy of COS7 cells 24, 36, 48, 72, and 96 h following transfection with 5′(CGG^exp^)-*GFP*(+1) construct and Hoechst 33342 staining. Insoluble fraction of FMRpolyG-GFP was calculated as the number of foci per 100 cells (*n* = 7). Note that up to 72 h post transfection the number of FMRpolyG-GFP foci per cell increases significantly reaching ∼10 per 100 cell, and then decreases due to cell division and cell death. Scale bar, 50 μm.

**FIGURE 3:**
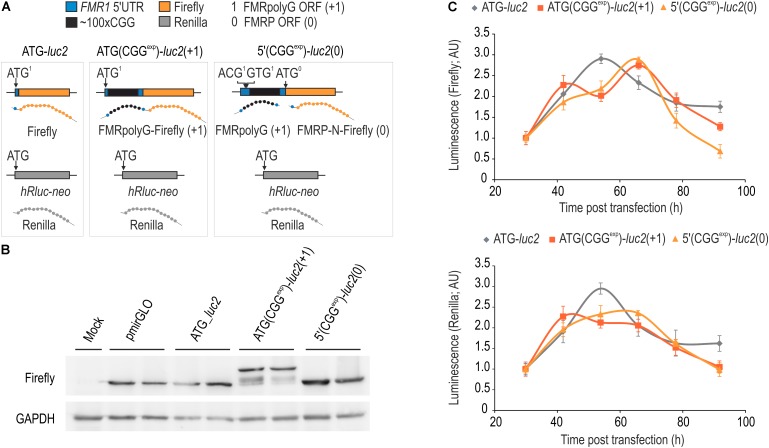
FMRpolyG biosynthesis from AUG-induced start codon can be assessed with luciferase assay. **(A)** Schematic representation of genetic constructs used in western blotting and luminescence assays. ATG(CGG^exp^)-*luc2*(+1) and 5′(CGG^exp^)-*luc2*(0) were used to compare basic dynamic properties of FMRpolyG-Firefly and FMRP-N-Firefly in relation to Firefly protein expressed from the control ATG-*luc2* construct. Renilla expressed independently from all constructs was used as an internal reference in luminescence assays. **(B)** Immunoblotting of COS7 cell extracts for Firefly and GAPDH 52 h post transfection with pmirGLO, ATG-*luc2*, ATG(CGG^exp^)-*luc2*(+1) or 5′(CGG^exp^)-*luc2*(0). Note correspondingly increased size of FMRpolyG-Firefly fusion protein in relation to Firefly and FMRP-N-Firefly. **(C)** Graphical representation of Firefly and Renilla luminescence signals from cell extracts prepared 30, 42, 54, 66, 78, and 96 h post transfection with ATG-*luc2*, ATG(CGG^exp^)-*luc2*(+1) or 5′(CGG^exp^)-*luc2*(0). Note similar Firefly and Renilla luminescence signals at different time points for both FMRpolyG-Firefly and FMRP-N-Firefly (*n* = 4).

**FIGURE 4:**
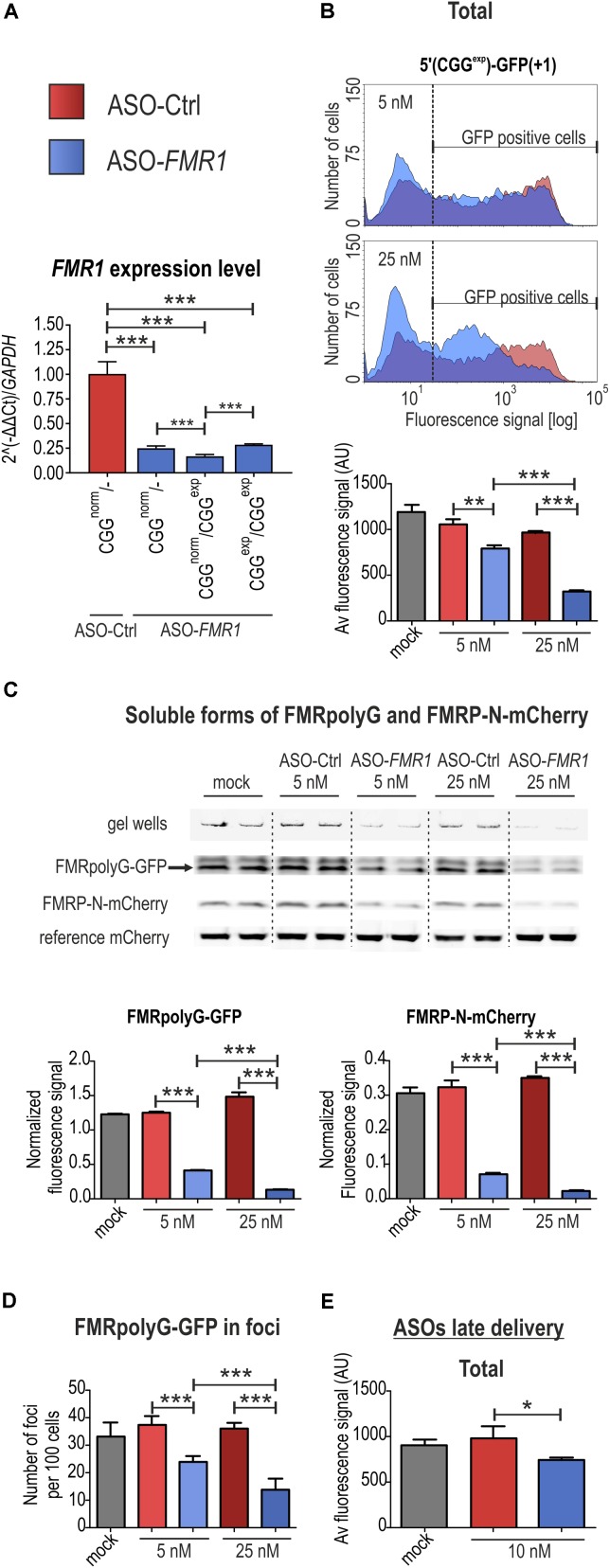
FMRpolyG fluorescence-based system can be used for efficient monitoring of therapeutic potential of molecules. **(A)** RT-qPCR analyses showing *FMR1* expression levels in FXTAS patient-derived fibroblasts after 48 h treatment with 10 nM control (ASO-Ctrl) or directed against *FMR1* antisense oligonucleotides (ASO-*FMR1*). Significant downregulation was observed in either control fibroblasts (CGG^norm^/-) or two fibroblast lines obtained from FXTAS patients: CGG^norm^/CGG^exp^ (mutant and normal allele), CGG^exp^/CGG^exp^ (two mutant alleles). All values are normalized to the level of *GAPDH* mRNA (*n* = 3). **(B)** Representative histograms showing distribution of FMRpolyG-GFP signals in COS7 cells 48 h post transfection with 5′(CGG^exp^)-*GFP*(+1) and early delivery of ASO-Ctrl or ASO-*FMR1* as well as quantification of the signals obtained from the flow cytometry experiments (lower panel). Note that ASO-*FMR1*, but not ASO-Ctrl, induced reduction of the number of cells showing high fluorescence and mean fluorescence signals in concentration dependent manner (*n* = 3 for each condition). **(C)** Quantitative FL-SDS-PAGE analysis of COS7 cells 48 h after co-transfection with 5′(CGG^exp^)-*GFP*(+1) and 5′(CGG^exp^)-*mCherry*(0) and early administration of either 5 or 25 nM ASO-Ctrl and ASO-*FMR1*. Delivery of ASO-*FMR1* at 25 nM concentration significantly decreased the amount of soluble FMRpolyG-GFP and FMRP-N-mCherry by ∼90%. Results were normalized to the reference mCherry signals derived from the control mCherry construct (*n* = 3). **(D)** Diagram showing quantification of foci numbers per 100 cells 48 h following 5′(CGG^exp^)-*GFP*(+1) administration and early ASO delivery (*n* = 6). Cells treated with 25 nM ASO-*FMR1* revealed a ∼60% decrease of FMRpolyG-GFP foci. **(E)** Quantification of the average fluorescence FMRpolyG-GFP signal in COS7 cells 72 h after transfection with 5′(CGG^exp^)-*GFP*(+1) and late delivery of 10 nM ASO-Ctrl or ASO-*FMR1* (*n* = 3). Note that 10 nM ASO-*FMR1* only slightly reduced the total FMRpolyG-GFP signal.

**FIGURE 5:**
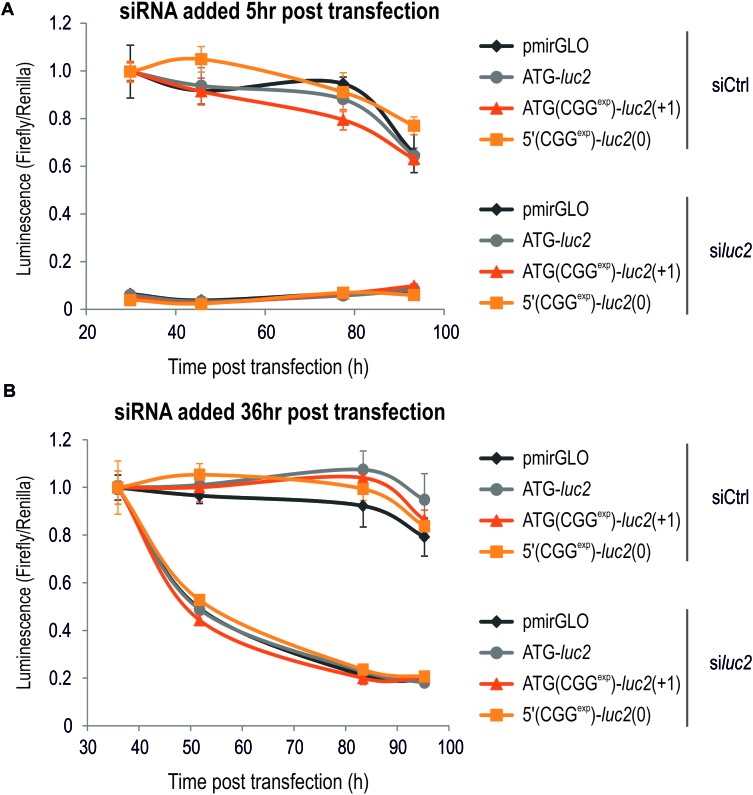
Luminescence assay can be efficiently used to monitor action of potential therapeutics. **(A,B)** Graphs showing luminescence Firefly signals in relation to the Renilla in COS7 cells transfected with various constructs as indicated in the figure and treated with siCtrl and si*luc2* delivered either 5 **(A)** or 36 **(B)** h post transfection. Note very efficient downregulation of the Firefly-based luminescence following si*luc2* administration, but not siCtrl (*n* = 4). No difference in the luminescence was observed for either Firefly or Renilla for any of the tested constructs.

**FIGURE 6:**
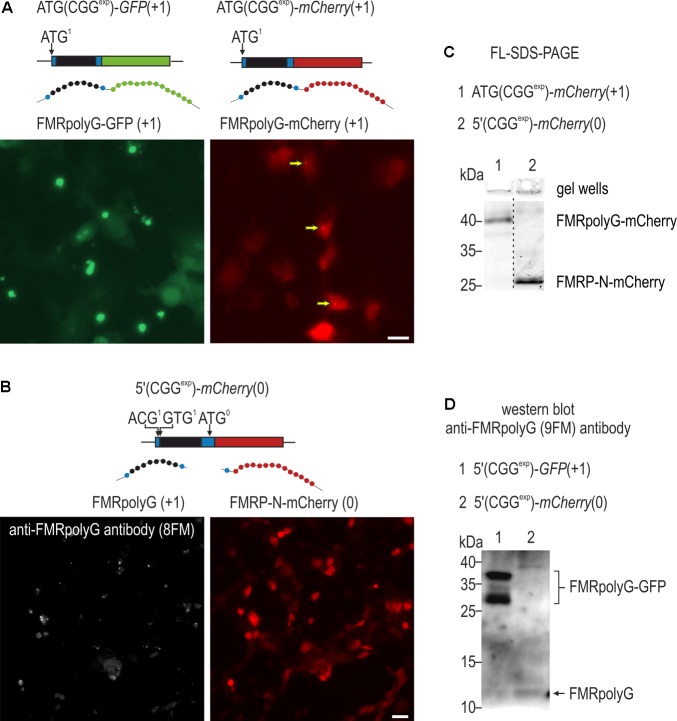
Tagging affects aggregation properties of FMRpolyG. **(A)** Fluorescence microscopy of COS7 cells 48 h post transfection with ATG(CGG^exp^)-*GFP*(+1) or ATG(CGG^exp^)-*mCherry*(+1). In contrast to FMRpolyG-GFP, FMRpolyG-mCherry forms only scarce foci (marked with arrows). **(B)** Visualization of COS7 cells 48 h following delivery of 5′(CGG^exp^)-*mCherry*(0) and labeling for FMRpolyG with 8FM antibody. Schematic representations of delivered constructs are shown above the images in **(A,B)**. Scale bars, 10 μm. **(C)** FL-SDS-PAGE analyses of soluble fractions of FMRpolyG-mCherry and FMRP-N-mCherry in cell lysates prepared 120 h after transfection with ATG(CGG^exp^)-*mCherry*(+1) or 5′(CGG^exp^)-*mCherry*(0). Note altered gel migration of the fusion protein in relation to FMRP-N-mCherry. Also, no increase in the FMRpolyG-mCherry signal is observed in gel wells, indicating absence of prominent protein foci. **(D)** Immunoblotting of COS7 cell lysates for FMRpolyG with 9FM antibody 48 h after administration of 5′(CGG^exp^)-*GFP*(+1) or 5′(CGG^exp^)-*mCherry*(0). Marked differences in the amounts of FMRpolyG-GFP and FMRpolyG are observed.

For average fluorescence signal quantification (**Figure 1B**) and total foci number and area estimation (Supplementary Figure S1), COS7 cells were seeded on a 96 well plate a day before transfection. ATG(CGG^exp^)-*GFP*(+1) and 5′(CGG^exp^)-*GFP*(+1) constructs were delivered with X-tremeGENE HP DNA Transfection Reagent (Roche; 125 ng/0.25 μl) when cells reached 40% confluency. In the case of *mCherry* constructs (**Figures 6A,B**), 160 ng of either ATG(CGG^exp^)-*mCherry*(+1) or 5′(CGG^exp^)-*mCherry*(0) was delivered with Lipofectamine 3000 according to the manufacturer’s instructions. For fluorescence microscopy following Hoechst staining (**Figures 2B**, >**4D**), COS7 cells were seeded on a 96 well plate and transfected with 125 ng of 5′(CGG^exp^)-*GFP*(+1) construct per well. For FL-SDS-PAGE (**Figures 2A**, **4C**) COS7 cells were seeded on a 48 well plate and co-transfected with 100 ng of 5′(CGG^exp^)-*GFP*(+1) construct, 100 ng of 5′(CGG^exp^)-*mCherry*(0) construct and 50 ng of control *mCherry* construct expressing MBNL-mCherry, i.e., reference mCherry (MB1-40-mCherry; Sznajder et al., 2016) per well. For flow cytometry (**Figures 1C**, **4B,E** and Supplementary Figures S2, S4) COS7 cells were seeded on 48 well plate and transfected with either 250 ng of 5′(CGG^exp^)-*GFP*(+1) construct or control construct expressing MBNL-GFP, (MBNL3-39-GFP; Sznajder et al., 2016) per well. For western blotting (**Figure 6D**) COS7 cells were seeded on a 6 well plate and transfected with either 2 μg of 5′(CGG^exp^)-*GFP*(+1) construct or 5′(CGG^exp^)-*mCherry*(0) construct. For fluorescence microscopy following Hoechst staining, FL-SDS-PAGE, flow cytometry and western blotting (**Figure 6D**) cells were transfected at 80–90% confluency using Lipofectamine 3000 (Thermo Fisher Scientific) according to manufacturer’s protocol, 2 h from plating. For luciferase assay (**Figures 3C**, **5A,B**) COS7 cells were grown on a 96 well plate and pmirGLO, ATG-*luc2*, ATG(CGG^exp^)-*luc2*(+1) or 5′(CGG^exp^)-*luc2*(0) constructs were delivered with X-tremeGENE HP DNA Transfection Reagent (Roche; 50 ng/0.1 μl). For immunoblotting, pmirGLO, ATG-*luc2*, ATG(CGG^exp^)-*luc2*(+1) or 5′(CGG^exp^)-*luc2*(0) constructs were delivered with X-tremeGENE HP DNA Transfection Reagent (0.4 μg/0.8 μl) to COS7 cells grown on a 12 well plate.

### ASOs and siRNA Delivery

Fifteen nucleotide-long ASOs included three LNA modified nucleotides at 3′ and 5′ ends and nine DNA nucleotides in the central core. All positions were phosphorothioated. ASOs were synthetized and purified by Kaneka Eurogentec. Fibroblast cell lines were seeded on 24 well plates and transfected with ASOs at 10 nM concentration, 12 h from plating. For fluorescence-based system experiments, COS7 cells were transfected with ASOs at 5, 10, or 25 nM concentration, 3 or 36 h from the construct delivery. ASOs were denaturated before transfection for 30 s in 95°C and delivered with Lipofectamine 3000, according to manufacturer’s protocol. For luciferase assays annealed siRNA oligos, control (*F4*/*R4*; siCtrl) and targeting luc2 (*F5*/*R5*; si*luc2*; Future Synthesis), were delivered to COS7 cells with Lipofectamine 2000 either 5 or 36 h post transfection with constructs according to the manufacturer’s protocol.

### Fluorescence Microscopy

Images were taken with Axio Observer.Z1 microscope equipped with AxioCam MRm camera, filter set 09 or 10 (GFP), 49 (Hoechst 33342) and 31 (mCherry), A-Plan 10×/0.25 Ph1 objective (Zeiss), and AxioVs40 module. For average fluorescence signal quantification (**Figure 1B**) and total foci number and area estimation (Supplementary Figure S1), images were taken 36, 48, 60, 72, 84 h post transfection from the same wells. Presented values were quantified from 10 images, each from a different well plate using ImageJ. Total fluorescence was estimated based on background subtraction (rolling ball algorithm) and mean intensity quantification. Inclusion areas and numbers were analyzed following image thresholding. Fluorescence microscopy following Hoechst staining were conducted 24, 36, 48, 72, or 96 h post transfection with constructs (**Figure 2B**) or 48 h post constructs and ASOs delivery (**Figure 4D**). Cells were washed in warm PBS and barely attached dead cells were removed by gentle shaking. Before analysis cells were incubated in standard growth medium with final concentration of 5 μg/ml of Hoechst 33342 (Thermo Fisher Scientific) for 30 min at 37°C. Number of cells and foci were calculated with ImageJ and 3d object counter plugin (Bolte and Cordelieres, 2006). For immunostaining (**Figure 6B**), COS7 cells were fixed for 15 min with 4% PFA 48 h post transfection and blocked for 1 h in 1% BSA diluted in PBS-Tween (0.1%; PBS-T). Incubation with mouse FMRpolyG 8FM primary antibody (1:50; see below) was conducted O/N at 4°C in the blocking solution. Secondary goat anti-mouse FITC-labeled antibody (1:400; Jackson ImmunoResearch Laboratories) was applied for 1 h at RT in PBS-T.

### Flow Cytometry

For flow cytometry, cells were harvested 24, 36, 48, 72, or 96 h post transfection with constructs (**Figure 1C** and Supplementary Figure S2), 48 h post constructs and ASO delivery (**Figure 4B** and Supplementary Figure S4), or 72 h post transfection with constructs and 36 h post ASOs delivery (**Figure 4E**). Cells were trypsinized, collected and centrifuged for 5 min at 300 g. Cell pellet was suspended in 100 μl of PBS, propidium ioide (PI) solution (Sigma) was added to final concentration of 1 μg/ml and incubated for 5 min at RT in darkness. Hundred microlliter of cell suspension was diluted with 150 μl of PBS and analyzed with guava easyCyte^TM^ HT System (Millipore). GFP and PI fluorescence was excited by blue laser (488 nm) and detected at 525/30 nm and 695/50 nm, respectively. For each sample ∼5000 GFP positive cells were collected. Threshold for GFP positive cells was set based on signal from non-transfected cells for which the percent of GFP positive cells was lower than 0.5%. PI negative cells were included for signal distribution and mean signal quantification.

### FL-SDS-PAGE

For FL-SDS-PAGE cells were harvested 24, 36, 48, 72, 96 h (**Figure 2A**) or 120 h (**Figure 6C**) post transfection with constructs or 48 h post constructs and ASOs delivery (**Figure 4D**). Cells were washed with ice cold PBS and 20 μl of lysis buffer (50 mM Tris–HCl, pH 7.4, 100 mM NaCl, 1% NP-40, 0.1% SDS, 0.5% sodium deoxycholate, benzamidine, PMSF) was added for each well. Plate was incubated on ice for 30 min. Cell layer was lifted using pipette tip and lysate was collected in a 1.5 ml tube. Samples were vortexed, sonicated for 5 cycles (30 s on/90 s off) using Bioruptor Plus (Diagenode) and frozen at -20°C. Five microlliter of total lysate was mixed with standard sample buffer (4×) and separated on a 12% SDS polyacrylamide gel (Mini-PROTEAN Tetra System, Bio-Rad) without heat-induced denaturation and centrifugation of sample. FMRpolyG-GFP dissolved in lysis buffer and resolved in the gel is considered a soluble fraction. The remaining portion, which could not be separated using SDS-PAGE is regarded as an insoluble FMRpolyG-GFP fraction. Gels were scanned using Amersham Typhoon RGB Biomolecular Imager. GFP and mCherry fluorescence was excited by 488 and 532 nm and detected using Cy2 and Cy3 filters, respectively. For visualization of PageRuler Prestained Protein Ladder, 10 to 180 kDa (Thermo Fisher Scientific) gel were scanned at 635 nm wavelength and detected using Cy5 filter. Images were analyzed with IQTL software.

### Western Blotting

For western blotting (**Figure 3B**), cells were harvested 52 h post transfection and processed as described before (Konieczny et al., 2017). Rabbit polyclonal antibody (PA5-32209; Thermo Fisher) and mouse monoclonal anti-human antibody (sc-47724; Santa Cruz) were used to detect Firefly luciferase and GAPDH, respectively. In Supplementary Figure S3A, FL-SDS-PAGE gels were used for protein transfer to pore-size 0.45 μm nitrocellulose membrane (Amersham) and immunoblotting. For western blot shown in **Figure 6D**, cells were lysed with 400 μl of lysis buffer (50 mM Tris–HCl, 150 mM NaCl, 1% NP-40, 0.1% SDS, 0.5% sodium deoxycholate, benzamidine, PMSF) 48 h post transfection and processed using the FL-SDS-PAGE protocol, with additional steps that included centrifugation at 13,000 rpm for 15 min at 4°C and heating for 5 min at 95°C before SDS-PAGE. Proteins were subsequently transferred to pore-size 0.2 μm PVDF membrane (Millipore), activated in methanol prior to use. Mouse monoclonal antibodies, 8FM and 9FM (a kind gift from N. Charlet-Berguerand; see also Buijsen et al., 2014), and secondary goat anti-mouse peroxidase conjugate antibody (31430, Thermo Fisher Scientific), were used to detect the N- and C-terminal parts of FMRpolyG. Images were captured using G:Box Chemi-XR5 (Syngene).

### Luciferase Assay

For luciferase assay (**Figures 3C**, **5A,B**) cells were harvested at different timepoints as indicated in figure legends. Following the lysis, the cells were transferred to a Nunc F96 MicroWell Black Polystyrene Plate (137101, Thermo Scientific) and luminescence of Firefly and Renilla luciferases were measured consecutively using Dual Luciferase Assay System (Promega), infinite F200 PRO, and i-control 1.8 SP1 microplate reader software (Tecan).

### RNA Isolation and RT-qPCR

Fibroblasts were harvested 48 h post ASOs delivery (**Figure 4A**). RNA was isolated with TRI Reagent (Sigma) according to the manufacturer’s instructions. Total RNA was reverse-transcribed with GoScript^TM^ Reverse Transcriptase (Promega) and random primers (Promega). Real-time quantitative PCR (qPCR) was performed with primers *F6/R6* for *FMR1*, *F7/R7* for *GAPDH* and 2× iTaq^TM^ universal SYBR^®^ Green supermix (Bio-Rad) and analyzed on a QuantStudio 7 Flex Real-Time PCR System machine.

### Statistical Analysis

Group data are expressed as the means ± standard deviation (SD); *n* = 3–10. The statistical significance was determined by unpaired two-tailed Student’s *t*-test using Statistica (^∗^ indicates *p* < 0.05; ^∗∗^ indicates *p* < 0.01 and ^∗∗∗^ indicates *p* < 0.001).

### Oligonucleotides

F1 5′-ATACTGACCATCAGTCCTAGGCTGGTGAGCAAGGGC
F2 5′-TAAGTCCCTGAGGCTTCTAGACTTACTTGTACAGC
F2 5′ P-TCGAGCCCGTCTAGAGGTCCCGGGACGCG (P, phosphorylation)
R2 5′ P-AATTCGCGTCCCGGGACCTCTAGACGGGC (P, phosphorylation)
F3 5′ ATCGCCTCGAGGGTGAGCAAGGGCGAGGAGGA
R3 5′ ATCGCTCTAGACTACAGCTCGTCCATGC
F4 5′ P-UAAGGCUAUGAAGAGAUAC*TT* (P, phosphorylation; RNA/*DNA*)
R4 5′ P-GUAUCUCUUCAUAGCCUUA*TT* (P, phosphorylation; RNA/*DNA*)
F5 5′ P-GGACGAGGACGAGCACUUC*TT* (P, phosphorylation; RNA/*DNA*)
R5 5′ P-GAAGUGCUCGUCCUCGUCC*TT* (P, phosphorylation; RNA/*DNA*)
F6 5′ ATCCCAACAAACCTGCCACA
R6 5′ ATGTGCTCGCTTTGAGGTGA
F7 5′ GAGTCAACGGATTTGGTCGT
R7 5′ TGATTTTGGAGGGATCTCG
  ASO-*FMR1* 5′ CTTCAGCCCTGCTAG (DNA/LNA)
  ASO-ctrl 5′ GTGACTAAGGTGCTA (DNA/LNA)

## Results

### Fluorescence-Based Systems to Monitor Biosynthesis and Aggregation of FMRpolyG

To evaluate both biosynthesis and aggregation of FMRpolyG we force expressed its fluorescent fusion version (FMRpolyG-GFP) in COS7 cells by delivering one of the two plasmids, differing in its expression rate (**Figure 1A**). 5′(CGG^exp^)-*GFP*(+1) contains the native 5′ untranslated region (5′UTR) of the *FMR1* gene with CGG^exp^ repeats preceded by the near cognate ACG triplet embedded in the Kozak sequence context. ATG(CGG^exp^)-*GFP*(+1) lacks the upstream *FMR1* 5′UTR sequence from the start codon and the ACG is replaced for ATG, which results in a robust FMRpolyG-GFP production (**Figure 1B**). Upon either of the plasmid delivery, we observed steadily increasing number, and the total area of foci over the course of the experiment (Supplementary Figures S1A,B). Additionally, in agreement with about 2.5 time higher overall fluorescence signal intensity starting from the 48 h time point (**Figure 1B**), the average inclusion size was markedly larger upon delivery of ATG(CGG^exp^)-*GFP*(+1) than 5′(CGG^exp^)-*GFP*(+1) (Supplementary Figures S1C,D).

Accumulation of FMRpolyG-GFP was accompanied by dispersed fluorescence signal (**Figure 1B** and Supplementary Figure S1C). To more precisely quantify its total level (both soluble and insoluble forms), we used flow cytometry-based detection system, which not only enabled measurement of the mean sample fluorescence intensity, but also signal distribution in a population of living cells. Upon delivery of 5′(CGG^exp^)-*GFP*(+1), the percentage of GFP positive cells increased until the 48 h time point (39%) while the number of cells showing the highest fluorescence content steadily increased throughout the experiment (**Figure 1C**, histograms). As the consequence, we observed a constant growth of the mean GFP signal until 72 h time point (**Figure 1C**, graph in the lower panel; Supplementary Figure S2). The discrepancy between the percentages of GFP positive cells and the relative high numbers of cells showing enhanced FMRpolyG-GFP level could be due to the plasmid loss during consequent cell divisions.

To assess the level of the soluble FMRpolyG-GFP fraction at different time points following transfection, we resolved undenatured protein extracts using SDS-PAGE and measured GFP fluorescence in relation to mCherry signal (FL-SDS-PAGE). Two different plasmids were co-delivered to COS7 cells, 5′(CGG^exp^)-*GFP*(+1) and 5′(CGG^exp^)-*mCherry*(0) (**Figure 1A**). In the latter, FMRP-N-mCherry was expressed from the FMRP reading frame, different from the reading frame of FMRpolyG. Such experimental design allowed us to monitor the dynamics of protein aggregation as well as lower the cost and speed up the procedure in comparison to the classic western blotting. The fluorescent band representing FMRpolyG-GFP in FL-SDS-PAGE assay (**Figure 2A**) was additionally identified based on immunoblotting with anti-FMRpolyG antibodies (see Supplementary Figure S3). The analysis revealed significantly increased aggregation rate of FMRpolyG-GFP after 36 h, based on the decreased soluble form of FMRpolyG-GFP and its elevated total level estimated by flow cytometry. Some fraction of unresolved protein stacked in gel wells; however, the measure of this signal is not quantitative (**Figure 2A**). To more accurately assess fully aggregated form of FMRpolyG-GFP in foci, we delivered 5′(CGG^exp^)-*GFP*(+1) to COS7 cells and stained them with Hoechst prior to fluorescence microscopy (**Figure 2B**). The analysis revealed increasing amounts of foci in living cells until the 72 h time point, when the number of foci per 100 cells reached ∼10 (**Figure 2B**, right panel).

### Luciferase Assay Enables Assessment of FMRpolyG Production From an AUG Start Codon

We generated ATG(CGG^exp^)-*luc2*(+1) and 5′(CGG^exp^)-*luc2*(0) (**Figure 3A**) and delivered them with pmirGLO and ATG-*luc2* constructs to COS7 cells, to supplement the fluorescence data and evaluate the dynamics of Firefly-tagged FMRpolyG (FMRpolyG-Firefly) in relation to Firefly alone generated from the FMRP reading frame (FMRP-N-Firefly). Importantly, each of the plasmids has *hRluc-neo* sequence, from which a second luciferase, Renilla, is expressed independently and can be used as an internal normalization control. As predicted, immunoblotting with anti-Firefly luciferase antibody revealed expression of FMRpolyG-Firefly fusion protein from ATG(CGG^exp^)-*luc2*(+1) plasmid, while only Firefly was detected upon administration of either 5′(CGG^exp^)-*luc2*(0) or pmiGLO and ATG-*luc2* control constructs (**Figure 3B**). Unexpectedly, immunoblotting revealed a similar amount of protein for all tested constructs, which indicates that FMRpolyG-Firefly is well dissolved in the cell lysis buffer. In contrast to the fluorescence data (**Figure 1**), the luciferase assay revealed a steep signal decline following the 66 h time point for both the Firefly and Renilla and no significant difference in luminescence for FMRpolyG-Firefly and FMRP-N-Firefly was noted (**Figure 3C**).

### Changes in Biosynthesis and Aggregation of FMRpolyG Can Be Efficiently Monitored With Fluorescence- and Luminescence-Based Systems

We tested the fluorescence- and luminescence-based methods to address their utility for studying therapeutic potential of compounds targeting either mutated *FMR1* transcripts or the aggregation/stability of FMRpolyG. Based on the results described above, we chose two different time points following transfection for the compound delivery, ∼3 h to test the effect on FMRpolyG translation abrogation and 36 h to check the protein stability once FMRpolyG production was attenuated.

First, we tested the utility of antisense oligonucleotides (ASOs) carrying 5′ and 3′ LNA-modified ends. Following efficient downregulation of *FMR1* transcript with an ASO complementary to a fragment in the 5′UTR of the *FMR1* gene downstream of the CGG^exp^ (ASO-*FMR1*), but not a control ASO (ASO-Ctrl), in FXTAS fibroblasts (**Figure 4A**), we delivered them to COS7 cells transfected with 5′(CGG^exp^)-*GFP*(+1). In the flow cytometry-based detection assay, the early ASO-*FMR1*, but not ASO-Ctrl delivery, resulted in a significant drop in the percentage of GFP-positive cells, an increase in the number of cells with low-fluorescence signal and the reduction in the average fluorescence, in a concentration dependent manner (**Figure 4B**). This effect was specific as cells transfected with a GFP control plasmid reacted neither to the ASO-Ctrl nor ASO-*FMR1* administration (Supplementary Figure S4). Then, we evaluated ASO-*FMR1* activity in cells co-transfected with 5′(CGG^exp^)-*GFP*(+1), 5′(CGG^exp^)-*mCherry*(0) and a control construct expressing reference mCherry using the FL-SDS-PAGE assay, which allowed us to quantify FMRpolyG-GFP and FMRP-N-mCherry levels relative to the reference mCherry at the same time. After ASO-*FMR1* administration, we observed significant and concentration dependent reduction of both FMRpoly-GFP and FMRP-N-mCherry signals (**Figure 4C**). Finally, fluorescence microscopy showed that ASO-*FMR1* significantly reduced the number of FMRpolyG-GFP foci (**Figure 4D**).

Late ASO-*FMR1* delivery to cells transfected with 5′(CGG^exp^)-*GFP*(+1) resulted in only a small decrease of the mean fluorescence signal (**Figure 4E**). We concluded that the GFP-based system is less potent for evaluation of reduction of FMRpolyG-GFP content in cells in this experimental design, possibly due to the stabilization effect of the GFP tag (Todd et al., 2013; Konieczny et al., 2017; Sellier et al., 2017). To further address this, we delivered to COS7 cells ATG(CGG^exp^)-*luc2*(+1), 5′(CGG^exp^)-*luc2*(0) or ATG-*luc2* constructs and measured luminescence at different time points after early and late siRNA delivery (**Figure 5**). Importantly, siRNA directed against *luc2* (si*luc2*) almost completely abrogated Firefly translation, while no such effect was observed for the control compound (siCtrl; **Figure 5A**). Furthermore, si*luc2* administration 36 h post transfection also resulted in a marked Firefly luminescence drop (**Figure 5B**). This effect was observed independently of the construct, indicative of high solubility of FMRpolyG-Firefly.

### Tagging Affects FMRpolyG Properties

The different outcome of the fluorescence and luminescence-based results pointed toward a significant influence of tagging on FMRpolyG properties, such as stability, aggregation or solubility. To further address this, we generated ATG(CGG^exp^)-*mCherry*(+1) plasmid and delivered it to COS7 cells, comparing the fluorescent signal to that obtained after administration of ATG(CGG^exp^)-*GFP*(+1) (**Figure 6A**). As a point of reference, we visualized untagged FMRpolyG in cells transfected with 5′(CGG^exp^)-*mCherry*(0) and immunostained with an 8FM antibody recognizing the N-terminal part of the protein (**Figure 6B**). Importantly, while FMRpolyG and FMRpolyG-GFP formed prominent inclusions, we hardly observed FMRpolyG-mCherry aggregated in foci (**Figure 6A**; arrows). In accordance, there was no change in the amount of insoluble form of FMRpolyG-mCherry stacked in gel wells over FMRP-N-mCherry in FL-SDS-PAGE (**Figure 6C**). Previous data pointed to increased stability of GFP-tagged FMRpolyG (Todd et al., 2013; Sellier et al., 2017). To confirm this, we transfected COS7 cells with 5′(CGG^exp^)-*GFP*(+1) and 5′(CGG^exp^)-*mCherry*(0) and performed immunoblotting of cell lysates with 9FM antibody, directed toward the C-terminus of FMRpolyG (**Figure 6D**). The analysis revealed markedly higher content of FMRpolyG-GFP when compared to the untagged protein version.

## Discussion

We aimed at selecting the most suitable method for easy evaluation of therapeutic potential of compounds targeting either translation or inclusion formation of FMRpolyG. We decided to base our selection on a simple COS7 cell culture system, which allowed us to force express differently tagged FMRpolyG as well as to compare its dynamic properties to the protein expressed from the FMRP-specific open reading frame. The potentially therapeutic compounds were then delivered at two different time points post vector transfection, before and after FMRpolyG could be synthesized. Such established system enabled us to monitor the FMRpolyG signal over the course of a couple of days using fluorescence microscopy, flow cytometry, luminescence assay, FL-SDS-PAGE and western blotting. Although various constructs and methods have been employed in studying therapeutic aspects of FXTAS-related phenotypes prior to this work (Todd et al., 2013; Su et al., 2014; Yang et al., 2015, 2016; Sellier et al., 2017), our protocols enable direct assessment of protein aggregation and measurement of FMRpolyG biosynthesis dependent on their native biosynthesis mechanism as well as from the AUG-induced start codon. The latter may serve as a sensitive model for studying downregulation of FMRpolyG expression upon addition, for example, compounds binding to (CGG)^exp^.

The obvious advantage of our detection system is the relatively short time needed for testing potential therapeutics, as we can judge FMRpolyG inclusion formation in less than 2 days following plasmid delivery. This allows for fast, preliminary drug screening before using more time-consuming cell and mouse models. Specifically, foci were detected in 5–10% and 20–30% FXTAS neurons at 20 and 40 days of differentiation from iPS cells, respectively (Sellier et al., 2017). In animal models, approximately 25–30% cells had foci in 8-week old Tet-On doxycycline-inducible (Hukema et al., 2015) and 3-month old bigenic CMV-cre/full-length *FMR1* 5′UTR transgenic mice (Sellier et al., 2017), with higher amounts of cells containing foci in much later time points. Importantly, our system could be easily expanded to screen potential therapeutics for other RAN translation- and aggregate-related diseases, most of which are also late-onset, such as myotonic dystrophy, C9orf72 amyotrophic lateral sclerosis/frontotemporal dementia, spinocerebellar ataxias or Huntington disease (Green et al., 2016; Cleary and Ranum, 2017; Zhang and Ashizawa, 2017).

To our surprise, FMRpolyG properties varied depending on the attached tag. FMRpolyG-GFP formed vast inclusions, larger in the case of a strong ATG start codon, while only rare foci were noted when GFP was replaced for mCherry. Similar conclusions could be drawn from previously published reports (Todd et al., 2013; Oh et al., 2015), showing far fewer inclusions upon expression of mCherry than GFP (CGG)^exp^ constructs. Comparison of FMRpolyG-GFP to the untagged protein version revealed also that the latter forms prominent foci despite the relatively low protein content detected using western blot (**Figure 6**), suggesting differences in aggregation potential of these proteins. Moreover, the FMRpolyG-GFP inclusions were also very stable, partially irremovable upon addition of ASO targeting *FMR1* [see also (Hukema et al., 2015)]. In contrast, Firefly-based fusion was easily eliminated from the cell upon late addition of siRNA. Grounded on these results, we conclude that (1) addition of tags, particularly mCherry and Firefly luciferase, enhance solubility (diminish aggregation potential) of FMRpolyG and (2) FMRpolyG-GFP has greatly increased stability (**Figure 6**). Interestingly, despite its lower potential to form foci, FMRpolyG-mCherry sufficed for activation of the impairment of the ubiquitin proteasome system in HeLa cells (Oh et al., 2015). The differences in the properties of the FMRpolyG fusions could be attributed to altered steric properties of tagged proteins, the cytotoxicity of tags or their effect on transcription or translation (Liu et al., 1999; Baens et al., 2006; Shemiakina et al., 2012; Ansari et al., 2016; Ganini et al., 2017). It is important to note that although various cytotoxic properties of GFP have been observed, no differences in percentages of HeLa cells surviving forced expression of GFP and mCherry were noted (Shemiakina et al., 2012). Moreover, we did not see any significant differences in mortality between cells expressing FMRpolyG-GFP and FMRpolyG-mCherry. Future experiments should address the above possibilities, perhaps concentrating on conformational properties of different fusion proteins and their effect on stability, aggregation and localization of FMRpolyG.

Based on our results, GFP and luciferase tagging allows for fast and reliable evaluation of therapeutics targeting FMRpolyG biosynthesis either from native or the AUG-induced start codon. The GFP fusion protein appears at the same time suitable for assessment of FMRpolyG aggregation, by estimating the ratio of soluble and insoluble forms. To make screening assays more powerful and applicable in high- or medium-throughput format the isogenic lines having transgene under the control of chemically induced promoters, either stronger or weaker, could be established.

## Author Contributions

KS, MD, and PK: designed the study. MD, PK, KS, and AW: prepared the figures. MD: Data contribution (**Figures 1C**, **2**, **4B–E**, **6D** and Supplementary Figures S2–S4). PK: Data contribution (**Figures 1B**, **3**, **5**, **6A** and Supplementary Figure S1). AW: Data contribution (**Figures 6A–C**). MS: Data contribution (**Figure 4A**). PK, MD, and KS: wrote the manuscript.

## Conflict of Interest Statement

The authors declare that the research was conducted in the absence of any commercial or financial relationships that could be construed as a potential conflict of interest.
