# Supplementary material for: Quantitative Evaluation of Toxic Polyglycine Biosynthesis and Aggregation in Cell Models Expressing Expanded CGG Repeats
Source: Front Genet. 2018 Jun 19;9:216. doi: 10.3389/fgene.2018.00216 (PMC6018535; doi:10.3389/fgene.2018.00216)
Supplement: Supplementary file 1 [file Image_1.PDF]

Quantitative evaluation of toxic polyglycine biosynthesis and aggregation in cell models expressing expanded CGG repeats

Magdalena Derbis*, Patryk Konieczny*, Agnieszka Walczak, Michał Sekrecki and Krzysztof Sobczak[#]

Department of Gene Expression, Institute of Molecular Biology and Biotechnology, Adam Mickiewicz University, Umultowska 89, 61-614 Poznan, Poland

*these authors contributed equally to this work

[#] corresponding author ksobczak@amu.edu.pl

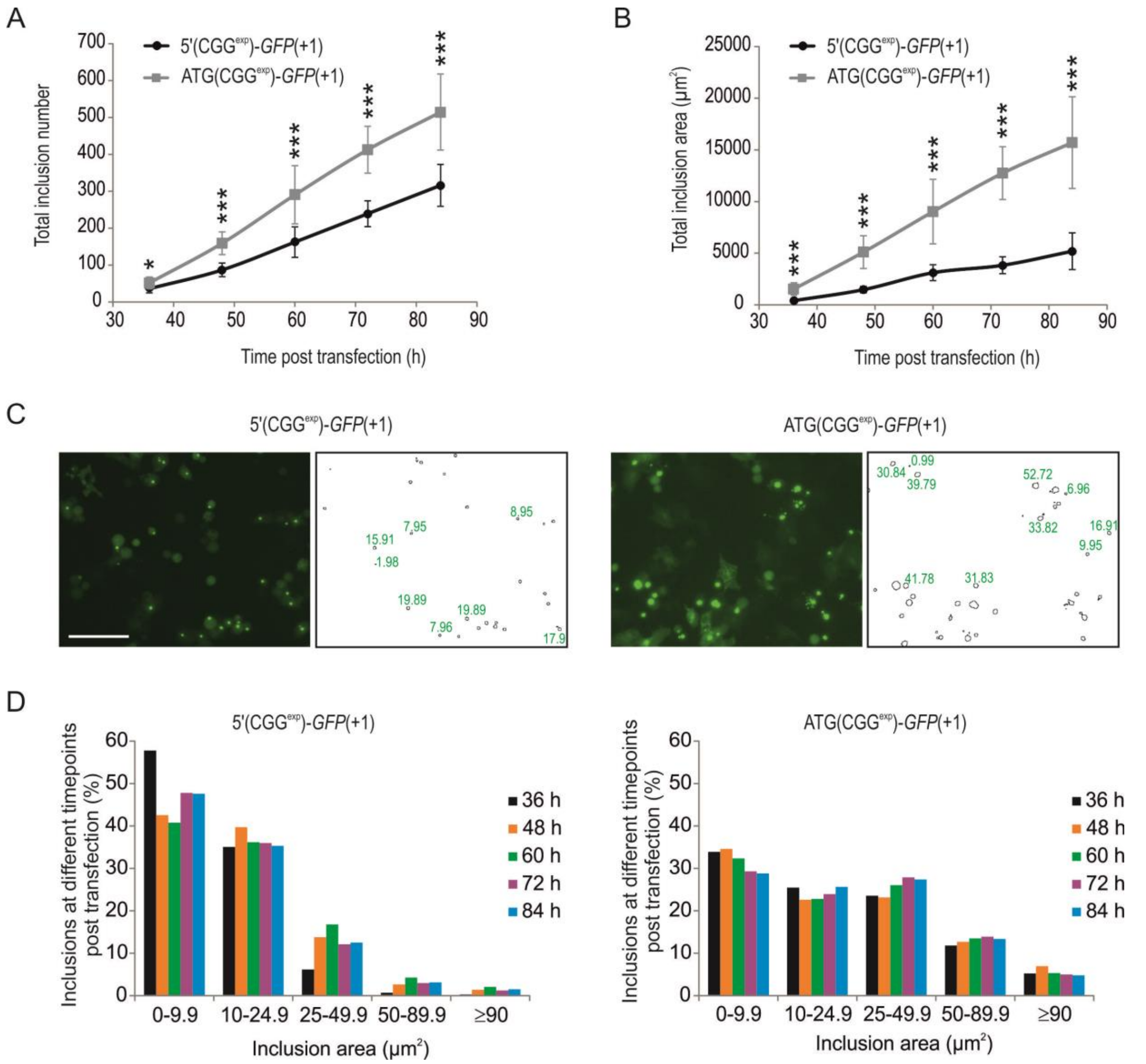

Figure S1. (related to Fig. 1B) **FMRpolyG-GFP inclusion numbers and areas depend on the translation rate. (A-B)** Graphs showing total inclusion numbers **(A)** and areas **(B)** 36, 48, 60, 72, and 84 h post transfection with either 5'(CGG^{exp})-*GFP*(+1) or ATG(CGG^{exp})-*GFP*(+1). Representative images used for quantifications are shown in Fig. 1B. Note markedly higher inclusion numbers and areas following transfection with ATG(CGG^{exp})-*GFP*(+1) than 5'(CGG^{exp})-*GFP*(+1); n=10. **(C)** Magnifications of the images shown in Fig. 1B at 72 h post transfection with either 5'(CGG^{exp})-*GFP*(+1) or ATG(CGG^{exp})-*GFP*(+1). Examples of inclusion outlines and areas (µm^2) are shown to the right of the magnified images. Scale bar, 100 µm. **(D)** Graphs showing percentages of inclusion areas, divided into five ranges as indicated in the figure, at 36, 48, 60, 72 and 84 h post transfection with either 5'(CGG^{exp})-*GFP*(+1) or ATG(CGG^{exp})-*GFP*(+1). Representative images used for quantifications are shown in Fig. 1B. Note, foci are markedly larger after transfection with ATG(CGG^{exp})-*GFP*(+1) than 5'(CGG^{exp})-*GFP*(+1).

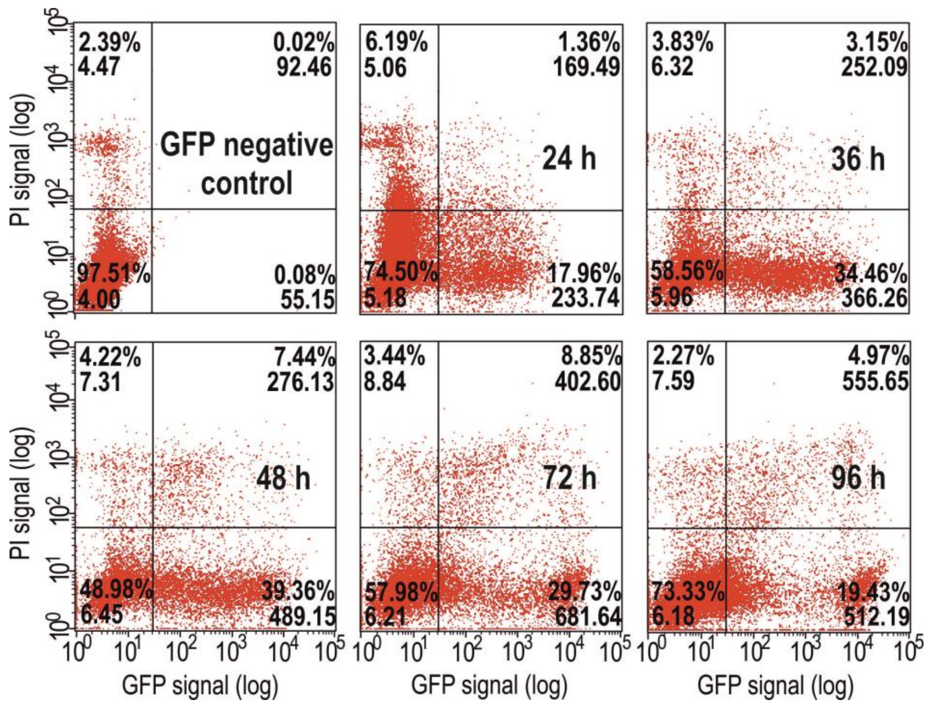

Figure S2. (related to Fig. 1C) **Flow cytometry dot plots.** Dot plot representation of histograms shown in Fig. 1C. Corner values indicate the mean fluorescence intensity and percentages of cells qualified as positive or negative in regard to PI and GFP fluorescence intensity.

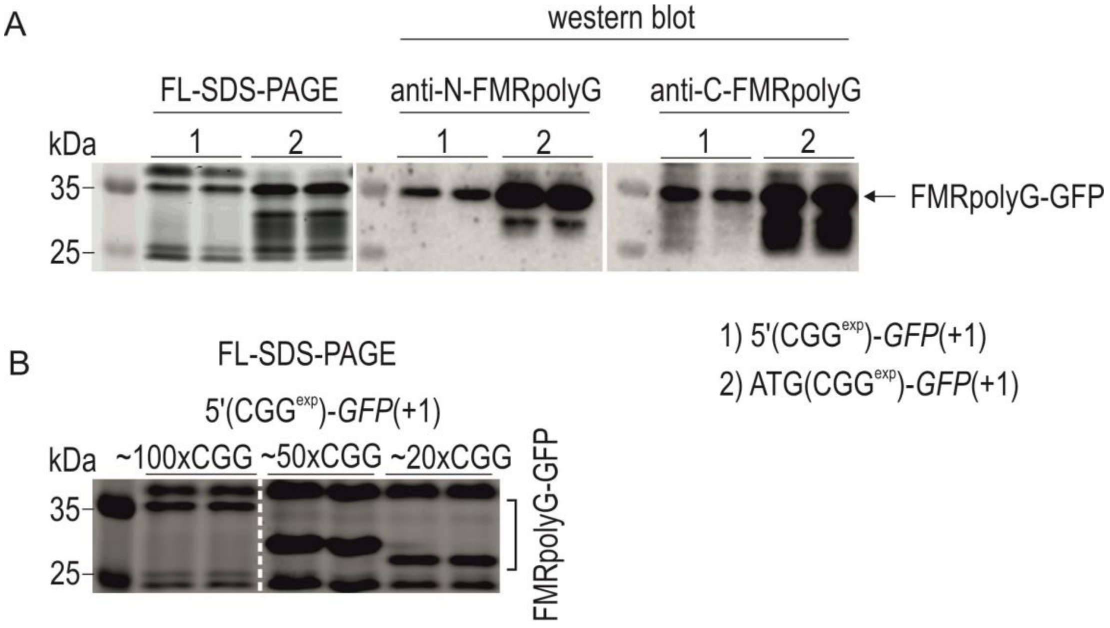

Figure S3. (related to Fig. 2) **Comparison of FL-SDS-PAGE and western blot FMRpolyG-GFP signals. (A)** Visualization of FMRpolyG with FL-SDS-PAGE and western blotting with antibodies against the N and C terminal parts of FMRpolyG as indicated in the figure. Cell lysates were prepared 48 h after transfection with either 5'(CGG^{exp})-*GFP*(+1) or ATG(CGG^{exp})-*GFP*(+1). The 35 kDa band was identified as FMRpolyG-GFP. Depending on the antibody used, there was four- or five-fold the amount of FMRpolyG-GFP in cell lysates following transfection with ATG(CGG^{exp})-*GFP*(+1) than 5'(CGG^{exp})-*GFP*(+1). **(B)** FMRpolyG-GFP visualization with FL-SDS-PAGE after transfection with 5'(CGG^{exp})-*GFP*(+1) carrying about 100, 50 and 20 CGG repeats. The band identified as FMRpolyG-GFP shows variable molecular weight depending on the construct variant.

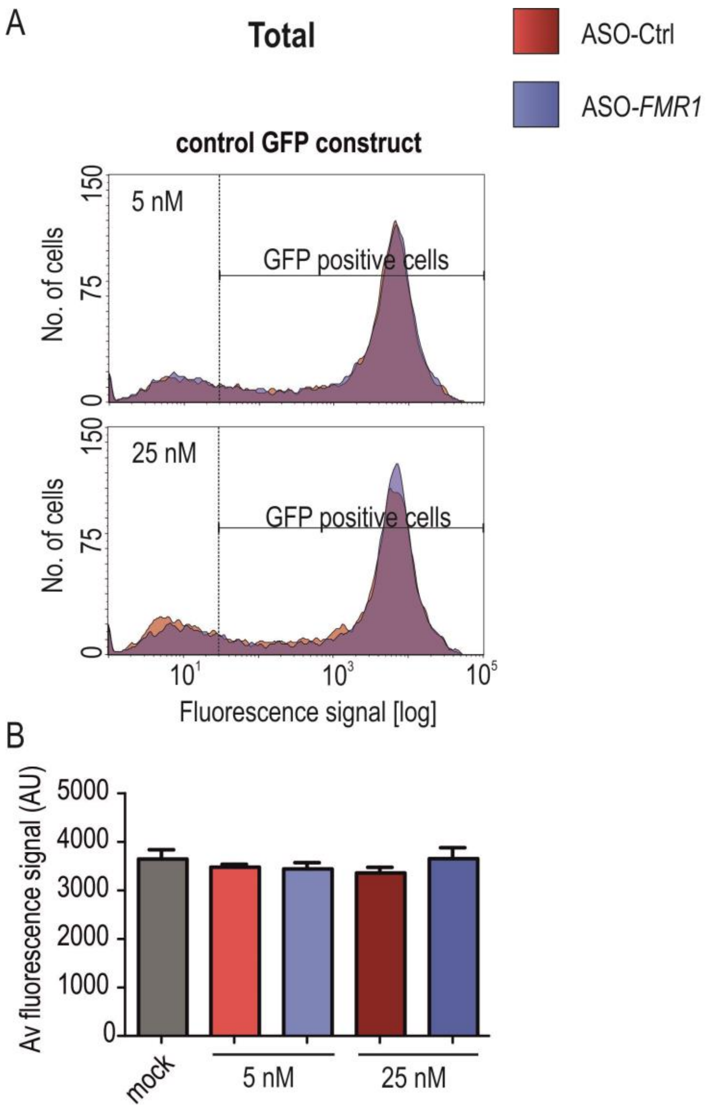

Figure S4. (related to Fig. 4) **Neither ASO-*FMR1* nor ASO-Ctrl change the mean fluorescence signal of cells transfected with control GFP construct. (A-B)** Histograms representing distribution of fluorescence FMRpolyG signal in population of cells transfected with control GFP construct and treated with either ASO-Ctrl or ASO-*FMR1* **(A)** and graphical representation of the obtained values **(B)**. Note that neither ASO-*FMR1* nor ASO-Ctrl change the mean fluorescence signal of cells transfected with control GFP construct (n=3).
